# Bis[*N*-(2-furylmeth­yl)ethane-1,2-di­amine]­bis­(perchlorato)copper(II)

**DOI:** 10.1107/S1600536811012232

**Published:** 2011-05-07

**Authors:** Wei Xiao, Shi-Rong Li, Hong Zhou, Zhi-Quan Pan, Qimao Huang

**Affiliations:** a Key Laboratory for Green Chemical Processes of the Ministry of Education, Wuhan Institute of Technology, Wuhan, 430073, People’s Republic of China; b Hubei Key Laboratory of Biologic Resources Protection and Utilization, Hubei Institute for Nationalities, Enshi, 44500, People’s Republic of China

## Abstract

In the title complex, [Cu(ClO_4_)_2_(C_7_H_12_N_2_O)_2_], the Cu(II) ion lies on a crystallographic inversion centre. The coordination sphere around Cu(II) ion can be described as tetragonally distorted octa­hedral with two perchlorate O atoms occupying the apical positions and four N atoms from two *N* ^1^-(2-furyl­methyl)ethane-1,2-diamine ligands in the basal plane.

## Related literature

For copper complexs with polyamine ligands, see: Souza *et al.* (2009 ▶); Patra *et al.* (2007 ▶); Zhou *et al.* (2009 ▶). For the synthesis, see: Wang *et al.* (2009 ▶).
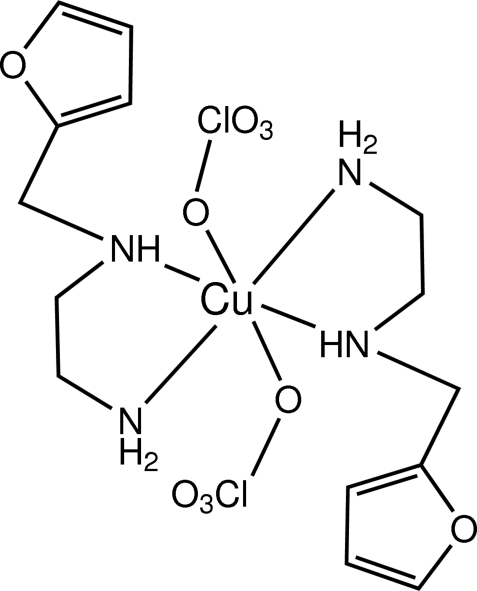


## Experimental

### 

#### Crystal data

[Cu(ClO_4_)_2_(C_7_H_12_N_2_O)_2_]
*M* *_r_* = 542.81
Monoclinic, 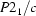

*a* = 9.736 (8) Å
*b* = 11.899 (9) Å
*c* = 9.466 (7) Å
β = 94.227 (12)°
*V* = 1093.6 (14) Å^3^
*Z* = 2
Mo *K*α radiation
μ = 1.30 mm^−1^
*T* = 291 K
0.28 × 0.24 × 0.22 mm

#### Data collection

Bruker SMART APEX CCD diffractometer
Absorption correction: multi-scan (*SADABS*; Sheldrick, 1996 ▶) *T*_min_ = 0.712, *T*_max_ = 0.763
5510 measured reflections
1914 independent reflections
1589 reflections with *I* > 2σ(*I*)
*R*_int_ = 0.039

#### Refinement

*R*[*F* ^2^ > 2σ(*F* ^2^)] = 0.049
*wR*(*F* ^2^) = 0.112
*S* = 1.07
1914 reflections
142 parameters
H-atom parameters constrained
Δρ_max_ = 0.26 e Å^−3^
Δρ_min_ = −0.70 e Å^−3^

### 

Data collection: *SMART* (Bruker, 2007 ▶); cell refinement: *SAINT* (Bruker, 2007 ▶); data reduction: *SAINT*; program(s) used to solve structure: *SHELXTL* (Sheldrick, 2008 ▶); program(s) used to refine structure: *SHELXTL*; molecular graphics: *SHELXTL*; software used to prepare material for publication: *SHELXTL*.

## Supplementary Material

Crystal structure: contains datablocks global, I. DOI: 10.1107/S1600536811012232/nk2094sup1.cif

Structure factors: contains datablocks I. DOI: 10.1107/S1600536811012232/nk2094Isup2.hkl

Additional supplementary materials:  crystallographic information; 3D view; checkCIF report

## Figures and Tables

**Fig. 1.**
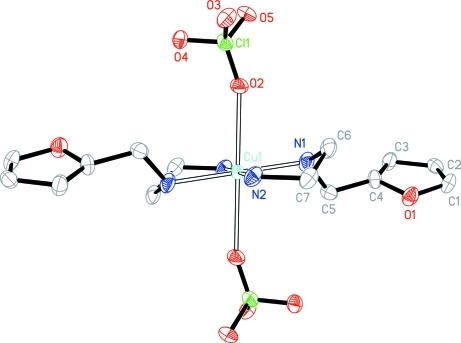
A view of the title complex cation, with displacement ellipsoids at the 30% probability level. H atoms are excluded for clarity. Unlabelled atoms are related to labelled atoms by inversion symmetry.

## supplementary crystallographic information

### Comment

Recently, study of copper complex with polyamine has been given considerable attention (Souza *et al.*, 2009; Patra *et al.*, 2007; Zhou *et al.*, 2009). In this paper, we report on the synthesis and the crystal structure determination of the title complex obtained by the reaction of Cu(ClO4)_2_6H_2_O with the polyamine ligand N^1^-(furan-2-ylmethyl)ethane-1,2-diamine.

In the title complex, [Cu(C_14_H_24_N_4_O_2_)_2_](ClO_4_)_2_, the Cu(II) ion lies on a crystallographic inversion centre. The coordination sphere around Cu(II) ion can be best described as slightly distorted octahedral. The basal plane is composed of four nitrogen atoms from the two polyamine ligands with the Cu-N distances of 2.001 (4) and 2.049 (4)Å. The apical positions are occupied by two oxygen atoms from two perchlorate anions with a Cu-O distance of 2.492 (4)Å.

### Experimental

N^1^-(furan-2-ylmethyl)ethane-1,2-diamine (L) was prepared according to the literature method (Wang *et al.*, 2009). Cu(ClO4)_2_6H_2_O (0.25 mmol, 0.093 g) dissolved in 10ml H2O was added dropwise to a solution of L (0.5 mmol, 0.071 g)in 10ml H2O. The mixture was stirred at ambient temperature for about 12 h and filtrated. The light blue crystals suitable for X-ray diffraction were obtained by the slow evaporation of the mother solution at ambient temperature for 3 weeks.

### Refinement

All H atoms for C-H distances were placed in calculated positions and included in the refinement in the riding-model approximation, with *U*(H) set to -1.2*U*_eq _of the parent atom.

### Crystal data

**Table:**

[Cu(ClO_4_)_2_(C_7_H_12_N_2_O)_2_]	*F*(000) = 558
*M**_r_* = 542.81	*D*_x_ = 1.648 Mg m^−^^3^
Monoclinic, *P*2_1_/*c*	Mo *K*α radiation, λ = 0.71073 Å
Hall symbol: -P 2ybc	Cell parameters from 1812 reflections
*a* = 9.736 (8) Å	θ = 2.7–22.6°
*b* = 11.899 (9) Å	µ = 1.30 mm^−^^1^
*c* = 9.466 (7) Å	*T* = 291 K
β = 94.227 (12)°	Block, blue
*V* = 1093.6 (14) Å^3^	0.28 × 0.24 × 0.22 mm
*Z* = 2	

### Data collection

**Table:**

Bruker SMART APEX CCD diffractometer	1914 independent reflections
Radiation source: sealed tube	1589 reflections with *I* > 2σ(*I*)
graphite	*R*_int_ = 0.039
ω scans	θ_max_ = 25.1°, θ_min_ = 2.7°
Absorption correction: multi-scan (*SADABS*; Sheldrick, 1996)	*h* = −9→11
*T*_min_ = 0.712, *T*_max_ = 0.763	*k* = −14→12
5510 measured reflections	*l* = −11→11

### Refinement

**Table:**

Refinement on *F*^2^	Primary atom site location: structure-invariant direct methods
Least-squares matrix: full	Secondary atom site location: difference Fourier map
*R*[*F*^2^ > 2σ(*F*^2^)] = 0.049	Hydrogen site location: inferred from neighbouring sites
*wR*(*F*^2^) = 0.112	H-atom parameters constrained
*S* = 1.07	*w* = 1/[σ^2^(*F*_o_^2^) + (0.05*P*)^2^ + 1.33*P*] where *P* = (*F*_o_^2^ + 2*F*_c_^2^)/3
1914 reflections	(Δ/σ)_max_ < 0.001
142 parameters	Δρ_max_ = 0.26 e Å^−^^3^
0 restraints	Δρ_min_ = −0.70 e Å^−^^3^

### Special details

**Table:**

Geometry. All e.s.d.'s (except the e.s.d. in the dihedral angle between two l.s. planes) are estimated using the full covariance matrix. The cell e.s.d.'s are taken into account individually in the estimation of e.s.d.'s in distances, angles and torsion angles; correlations between e.s.d.'s in cell parameters are only used when they are defined by crystal symmetry. An approximate (isotropic) treatment of cell e.s.d.'s is used for estimating e.s.d.'s involving l.s. planes.
Refinement. Refinement of *F*^2^ against ALL reflections. The weighted *R*-factor *wR* and goodness of fit *S* are based on *F*^2^, conventional *R*-factors *R* are based on *F*, with *F* set to zero for negative *F*^2^. The threshold expression of *F*^2^ > σ(*F*^2^) is used only for calculating *R*-factors(gt) *etc*. and is not relevant to the choice of reflections for refinement. *R*-factors based on *F*^2^ are statistically about twice as large as those based on *F*, and *R*- factors based on ALL data will be even larger.

### Fractional atomic coordinates and isotropic or equivalent isotropic displacement parameters (Å^2^)

**Table:**

	*x*	*y*	*z*	*U*_iso_*/*U*_eq_	
C1	0.4721 (5)	0.3096 (4)	0.7859 (4)	0.0440 (10)	
H1	0.3854	0.2781	0.7661	0.053*	
C2	0.5127 (4)	0.3583 (4)	0.9007 (5)	0.0421 (10)	
H2	0.4651	0.3609	0.9823	0.051*	
C3	0.6429 (5)	0.4080 (4)	0.8837 (5)	0.0476 (11)	
H3	0.6939	0.4531	0.9484	0.057*	
C4	0.6766 (4)	0.3770 (4)	0.7570 (4)	0.0404 (10)	
C5	0.8085 (4)	0.3823 (4)	0.6885 (4)	0.0417 (10)	
H5A	0.8831	0.3906	0.7616	0.050*	
H5B	0.8220	0.3116	0.6405	0.050*	
C6	0.7106 (5)	0.4843 (4)	0.4717 (5)	0.0468 (11)	
H6A	0.6259	0.4492	0.4970	0.056*	
H6B	0.6924	0.5634	0.4536	0.056*	
C7	0.7570 (5)	0.4316 (4)	0.3458 (5)	0.0466 (11)	
H7A	0.6909	0.4462	0.2662	0.056*	
H7B	0.7621	0.3509	0.3599	0.056*	
Cl1	0.90322 (11)	0.80490 (9)	0.47487 (11)	0.0422 (3)	
Cu1	1.0000	0.5000	0.5000	0.0320 (2)	
N1	0.8171 (4)	0.4721 (3)	0.5882 (4)	0.0465 (9)	
H1B	0.8199	0.5368	0.6377	0.056*	
N2	0.8951 (4)	0.4749 (3)	0.3128 (4)	0.0451 (9)	
H2A	0.8864	0.5396	0.2638	0.054*	
H2B	0.9387	0.4245	0.2611	0.054*	
O1	0.5768 (3)	0.3107 (3)	0.6947 (3)	0.0493 (8)	
O2	0.9102 (3)	0.6962 (3)	0.4956 (3)	0.0493 (8)	
O3	0.8334 (3)	0.8393 (2)	0.3418 (3)	0.0476 (8)	
O4	1.0257 (3)	0.8481 (2)	0.4616 (3)	0.0462 (7)	
O5	0.8431 (3)	0.8640 (2)	0.5750 (3)	0.0446 (7)	

### Atomic displacement parameters (Å^2^)

**Table:**

	*U*^11^	*U*^22^	*U*^33^	*U*^12^	*U*^13^	*U*^23^
C1	0.044 (3)	0.046 (2)	0.042 (2)	−0.018 (2)	0.0009 (19)	0.004 (2)
C2	0.041 (2)	0.045 (2)	0.042 (2)	0.0089 (19)	0.0103 (19)	0.0034 (19)
C3	0.048 (3)	0.049 (3)	0.048 (2)	−0.015 (2)	0.015 (2)	−0.009 (2)
C4	0.035 (2)	0.057 (3)	0.0306 (19)	−0.0051 (19)	0.0070 (17)	0.0011 (18)
C5	0.042 (2)	0.041 (2)	0.043 (2)	0.0078 (19)	0.0053 (19)	0.0011 (18)
C6	0.045 (3)	0.051 (3)	0.046 (2)	−0.006 (2)	0.012 (2)	0.019 (2)
C7	0.045 (3)	0.048 (3)	0.044 (2)	−0.012 (2)	−0.0140 (19)	0.012 (2)
Cl1	0.0397 (6)	0.0441 (6)	0.0442 (6)	0.0033 (4)	0.0113 (4)	−0.0060 (4)
Cu1	0.0324 (4)	0.0315 (4)	0.0333 (4)	−0.0005 (3)	0.0110 (3)	0.0024 (3)
N1	0.043 (2)	0.050 (2)	0.048 (2)	−0.0093 (17)	0.0127 (17)	0.0170 (17)
N2	0.043 (2)	0.043 (2)	0.048 (2)	−0.0126 (16)	−0.0073 (17)	−0.0004 (16)
O1	0.053 (2)	0.0482 (17)	0.0475 (17)	−0.0162 (15)	0.0109 (15)	−0.0149 (14)
O2	0.0527 (19)	0.0448 (18)	0.0530 (18)	0.0136 (14)	0.0218 (15)	0.0132 (14)
O3	0.0467 (17)	0.0419 (17)	0.0529 (18)	−0.0135 (14)	−0.0053 (14)	0.0056 (14)
O4	0.0469 (18)	0.0466 (17)	0.0474 (16)	−0.0021 (14)	0.0193 (14)	0.0084 (14)
O5	0.0430 (17)	0.0442 (16)	0.0467 (16)	0.0178 (14)	0.0040 (13)	−0.0015 (13)

### Geometric parameters (Å, °)

**Table:**

C1—C2	1.268 (6)	C6—H6B	0.9700
C1—O1	1.384 (5)	C7—N2	1.494 (6)
C1—H1	0.9300	C7—H7A	0.9700
C2—C3	1.419 (6)	C7—H7B	0.9700
C2—H2	0.9300	Cl1—O2	1.309 (3)
C3—C4	1.319 (6)	Cl1—O4	1.313 (3)
C3—H3	0.9300	Cl1—O5	1.348 (3)
C4—O1	1.352 (5)	Cl1—O3	1.446 (3)
C4—C5	1.482 (6)	Cu1—N2	2.001 (4)
C5—N1	1.436 (5)	Cu1—N2^i^	2.001 (4)
C5—H5A	0.9700	Cu1—N1	2.049 (4)
C5—H5B	0.9700	Cu1—N1^i^	2.049 (4)
C6—C7	1.448 (6)	N1—H1B	0.9000
C6—N1	1.465 (6)	N2—H2A	0.9000
C6—H6A	0.9700	N2—H2B	0.9000
			
C2—C1—O1	109.5 (4)	N2—C7—H7B	109.4
C2—C1—H1	125.3	H7A—C7—H7B	108.0
O1—C1—H1	125.3	O2—Cl1—O4	111.3 (2)
C1—C2—C3	108.5 (4)	O2—Cl1—O5	115.5 (2)
C1—C2—H2	125.8	O4—Cl1—O5	107.9 (2)
C3—C2—H2	125.8	O2—Cl1—O3	115.2 (2)
C4—C3—C2	105.7 (4)	O4—Cl1—O3	100.24 (19)
C4—C3—H3	127.1	O5—Cl1—O3	105.3 (2)
C2—C3—H3	127.1	N2—Cu1—N2^i^	180.000 (1)
C3—C4—O1	109.9 (4)	N2—Cu1—N1	86.25 (16)
C3—C4—C5	131.9 (4)	N2^i^—Cu1—N1	93.75 (16)
O1—C4—C5	116.9 (3)	N2—Cu1—N1^i^	93.75 (16)
N1—C5—C4	114.6 (4)	N2^i^—Cu1—N1^i^	86.25 (16)
N1—C5—H5A	108.6	N1—Cu1—N1^i^	180.0
C4—C5—H5A	108.6	C5—N1—C6	120.0 (4)
N1—C5—H5B	108.6	C5—N1—Cu1	119.0 (3)
C4—C5—H5B	108.6	C6—N1—Cu1	105.4 (3)
H5A—C5—H5B	107.6	C5—N1—H1B	107.1
C7—C6—N1	109.3 (4)	C6—N1—H1B	107.4
C7—C6—H6A	109.8	Cu1—N1—H1B	94.6
N1—C6—H6A	109.8	C7—N2—Cu1	106.0 (3)
C7—C6—H6B	109.8	C7—N2—H2A	110.5
N1—C6—H6B	109.8	Cu1—N2—H2A	110.5
H6A—C6—H6B	108.3	C7—N2—H2B	110.5
C6—C7—N2	111.2 (4)	Cu1—N2—H2B	110.5
C6—C7—H7A	109.4	H2A—N2—H2B	108.7
N2—C7—H7A	109.4	C4—O1—C1	105.8 (3)
C6—C7—H7B	109.4		
			
O1—C1—C2—C3	7.7 (5)	N2—Cu1—N1—C5	121.4 (3)
C1—C2—C3—C4	−4.6 (5)	N2^i^—Cu1—N1—C5	−58.6 (3)
C2—C3—C4—O1	−0.5 (5)	N2—Cu1—N1—C6	−16.7 (3)
C2—C3—C4—C5	−166.5 (5)	N2^i^—Cu1—N1—C6	163.3 (3)
C3—C4—C5—N1	−102.5 (6)	C6—C7—N2—Cu1	36.2 (4)
O1—C4—C5—N1	92.3 (5)	N1—Cu1—N2—C7	−9.8 (3)
N1—C6—C7—N2	−53.2 (5)	N1^i^—Cu1—N2—C7	170.2 (3)
C4—C5—N1—C6	−54.1 (5)	C3—C4—O1—C1	4.9 (5)
C4—C5—N1—Cu1	173.9 (3)	C5—C4—O1—C1	173.3 (4)
C7—C6—N1—C5	−96.6 (5)	C2—C1—O1—C4	−7.9 (5)
C7—C6—N1—Cu1	41.1 (4)		

Symmetry codes: (i) −*x*+2, −*y*+1, −*z*+1.
